# Sex, executive function, and prospective memory regulate the chain-mediation pathway of alcohol use and impulsivity

**DOI:** 10.3389/fpubh.2023.1292422

**Published:** 2023-12-20

**Authors:** Fan Duan, Lingling Xia, Junda Li, Xiangyu Li, Yiding Zhou, Hanrun Luo, Ze Wang, Xun Song, Jingjing Wang, Jinxuan Chen, Yue Wang, Jing Zhang, Xiaochu Zhang, Dongliang Jiao

**Affiliations:** ^1^School of Mental Health, Bengbu Medical College, Bengbu, Anhui, China; ^2^CAS Key Laboratory of Brain Function and Disease and School of Life Sciences, University of Science and Technology of China, Hefei, Anhui, China

**Keywords:** alcohol use disorder, impulsivity, executive function, prospective memory, chain-mediation pathway analysis

## Abstract

**Objective:**

Evidence from previous studies indicates that impulsive behaviors are closely linked to alcohol use and misuse and that female drinkers are more impulsive than male drinkers. However, studies investigating the psychological mechanisms of alcohol use and impulsivity based on sex differences are relatively limited.

**Methods:**

This cross-sectional study comprised 713 residents from 16 cities in Anhui Province, China. Each subject was evaluated for self-reporting measures using several questionnaires, including the general information questionnaire, the Alcohol Use Disorders Identification Test (AUDIT), the Prospective and Retrospective Memory Questionnaire (PRM), the Behavior Rating Inventory of Executive Function-Adult Version (BRIEF-A), and the Barratt Impulsiveness Scale-11 (BIS-11).

**Results:**

Executive function and prospective memory may serve as intermediary links between alcohol use and impulsivity. Although the female alcohol usage level was significantly lower than that of males, the female drinkers had more severe executive dysfunction, prospective memory impairment, and impulsivity than male drinkers. Sex moderated the relationship between alcohol use and impulsivity. Furthermore, the indirect effect of executive function, and prospective memory between AUDIT and BIS was more significant in males than in females.

**Conclusion:**

Alcohol consumption may be associated with impulsivity formation through executive dysfunction and PM impairment, implying that impulsivity in those with AUD or at risk for AUD might be treated by improving EF and PM. Alcohol use may cause more severe executive dysfunction, PM impairment, and impulsive behavior in females than in males, and impulsive behavior in women with AUD was more likely to be due to the direct effects of alcohol consumption, while impulsive behavior in men with AUD was more likely to be due to the indirect effects of executive dysfunction and PM impairment. These findings provide both clinical and theoretical foundations for addressing issues related to alcohol use.

## 1 Introduction

Alcohol Use Disorder (AUD) is a medical condition characterized by frequent and excessive alcohol consumption. This addiction disorder has persistently posed a significant social, health, and economic challenge. There has been a noticeable rise in the incidence of alcohol misuse among females in recent years. Therefore, understanding the potential mechanisms underlying alcohol use and how it varies by sex is crucial to developing new and more targeted therapeutic approaches (1).

Females progress more rapidly from the first alcohol intake to addiction or an illness than males (1–3). However, the number of studies investigating the psychological mechanisms of the effects of alcohol use based on sex differences is relatively limited. Impulsive behaviors are intimately associated with alcohol use and misuse, both as contributors and consequences of use. Drinkers with impulsive characteristics are more likely to relapse. Conversely, the acute and chronic effects of drug use may increase impulsive behaviors, facilitating further drug use (4, 5). Previous research reported that female heavy drinkers were more impulsive than their male counterparts (6, 7). Interestingly, the non-alcohol-seeking control males were similar or more impulsive than the female controls in these studies, implying a strong correlation between impulsive behavior and drug misuse among females than males. Consequently, exploring the psychological mechanisms between impulsivity and alcohol use is of great significance in AUD treatment (8).

Impulsivity is a concept that encompasses a wide range of poorly conceived, prematurely expressed, excessively risky, or situationally inappropriate actions that often result in undesirable outcomes (9). Growing evidence from preclinical laboratory animal trials and clinical studies indicates that impulsive behavior might be causally linked to multiple distinct drug addiction processes, including onset, maintenance, and relapse (10). However, the psychological mechanism underlying alcohol use and impulsivity remains unclear.

In 1995, Barratt and colleagues (11) defined impulsivity as swift, unplanned reactions without considering potential negative consequences. They posited that impulsivity encompasses three dimensions: “motor impulsiveness,” “non-planning impulsiveness,” and “attentional impulsiveness.” To measure impulsivity, they introduced the Barratt Impulsiveness Scale (BIS), which has since become a crucial tool worldwide. This study measured impulsivity in people who use or do not use alcohol, and measured the correlation between impulsivity and alcohol use severity. Thus, we utilized this scale to investigate impulsive behavior.

Based on its definition and composition, impulsivity may be associated with executive dysfunction and Prospective Memory (PM) impairment. Executive Functions (EFs) include abstract thinking, motivation, planning, attention to tasks, and suppression of impulsive responses. AUD interventions such as naltrexone pharmacotherapy and psychotherapy may exert therapeutic effects by enhancing EFs (5). On the other hand, PM is an ability to execute future intended actions, and its impairment could aggravate impulsivity (12). As a result, it can be deduced that executive dysfunction and PM impairment in those with AUD or at risk for AUD could promote impulsive behavior by weakening inhibitory control and disrupting the ability to execute future intended actions. According to previous research, executive dysfunctions (failures in planning, set-shifting, selective attention, or working memory) are involved in PM deficits (13–15). Both executive dysfunction and PM impairment in those with AUD or at risk for AUD may result from alcohol-misuse-induced brain structural and functional damage (16, 17). Moreover, evidence indicates that both sex and sex-related factors interact with AUD complexly, differentially impacting the risk of developing behavioral or medical problems in male and female drinkers (18).

Based on the above-mentioned findings, we hypothesized that EF and PM may play a chain intermediary role between alcohol use and impulsivity, and sex factors may differently affect EF and PM, leading to varied impulsivity outcomes in alcohol-dependent individuals. The findings of this study may offer a theoretical or clinical reference for AUD treatment.

## 2 Materials and methods

### 2.1 Participants and procedure

The researchers randomly selected a number of neighbourhoods with greater than 2,000 households in 16 cities in Anhui Province, China, between 3 July and 25 August 2021, and collected questionnaires within the neighbourhoods using a convenience sampling method. During the study, an online questionnaire survey method was employed, and only one questionnaire could be completed per IP address. 713 valid questionnaires were included in the final analysis. This study was approved by the Bengbu Medical College Institutional Review Board (Approval number: 2019–199), and all investigations complied with the regulatory approval.

### 2.2 Sample selection

The respondents who met the following criteria were included: (1) age above 18; (2) signed informed consent; (3) normal eyesight and hearing. The exclusion criteria were: (1) serious physical illness or mental illness (e.g., schizophrenia, affective disorders, epilepsy, or Parkinson’s disease); (2) dependence on substances besides alcohol (e.g., heroin, morphine and methamphetamine); (3) pregnancy; (4) alcohol allergy or medical advice not to drink alcohol.

### 2.3 Research tools

#### 2.3.1 General information questionnaire

The general questionnaire included variables such as sex, age, education, marriage, residence based on the needs of the study.

#### 2.3.2 Alcohol use disorders identification test (AUDIT)

The Chinese version of AUDIT with ten items was used to estimate drinking severity (19). A scoring system suitable for the Chinese population was used following recommendations in the literature (19, 20). Total scores ranged from 0 to 40; scores >7 and > 16 indicated risky drinking and alcohol dependence, respectively. The Cronbach’s α of the standardized scale item and the KMO test coefficient (Bartlett’s test, *p* < 0.05) were 0.838 and 0.906, respectively, indicating that the scale had excellent reliability and validity.

#### 2.3.3 Prospective and retrospective memory questionnaire

The Prospective and Retrospective Memory Questionnaire (PRM), a scale designed to provide a self-report measure of prospective and retrospective memory failures in everyday life, was previously translated into Chinese and validated by Chinese researchers (21). The scale encompassed both prospective and retrospective memory. In particular, retrospective memory was regarded as the foundation for the execution of prospective memory tasks. Hence, the combined assessment of these two forms of memory gauged the individual’s prospective memory proficiency. It comprises 16 items, eight each for PM and retrospective memory. The Cronbach’s α coefficients of the standardized item were 0.820 and 0.824 for PM and retrospective memory, respectively, and the total scale score was 0.901, indicating that the scale had excellent reliability and validity.

#### 2.3.4 Behavior rating inventory of executive function-adult version

The Chinese version of the Behavior Rating Inventory of Executive Function-Adult Version (BRIEF-A) developed by Roth et al. (22) was used to measure EF. It contains 75 items yielding an overall score and the Global Executive Composite (GEC) derived from two index scores [Behavioral Regulation Index (BRI) and Metacognitive Index (MI)]. The BRI comprises four clinical scales (Inhibit, Shift, Emotional Control, and Self-Monitor), whereas the MI comprises five clinical scales (Initiate, Working Memory, Plan or Organize, Task Monitor, and Organization of Materials). A 1–3 level scoring system with a score of 1 for “never,” a score of 2 for “sometimes,” and a score of 3 for “often” was adopted. The higher the total score, the more severe the EF impairment. The internal consistency Cronbach’s α of this scale and the KMO test coefficient (Bartlett’s test, *p* < 0.05) were 0.958 and 0.956, respectively, indicating that the scale had excellent reliability and validity.

#### 2.3.5 Barratt impulsiveness Scale-11 (BIS-11)

The Barratt Impulsiveness Scale-11 (BIS-11) (11) evaluates the impulsive characteristics of individuals. The Chinese version of the BIS-11 contains 30 questions divided into three dimensions: Unplanned impulsivity, Action impulsivity, and Cognitive impulsivity. Each dimension contains ten questions. Among them, action impulsivity is a positive item, corresponding to the 1 (never) to 5 (always) score range, while unplanned impulsivity and cognitive impulsivity are reverse items. The higher the total score, the more impulsive the individual. The internal consistency Cronbach’s α of this scale and the KMO test coefficient (Bartlett’s test, *p* < 0.05) were 0.765 and 0.914, respectively, indicating that the scale had good reliability and validity.

### 2.4 Statistical analysis

Mplus 8.3 was employed for conducting confirmatory factor analysis to assess the validity of variables. Given that data was non-normal distributed, Mplus Maximum Likelihood robust (MLr) was employed as parameter estimator (23). And SPSS 25.0 software was utilized for the statistical analysis in this study. The measured data were presented as median (interquartile range). To evaluate statistical differences among different groups, a Wilcoxon rank sum test was applied for variables with two categories, and a Kruskal-Wallis rank sum test was used for variables with three or more categories. Common method bias was examined using Harman’s single-factor test. Partial correlation analysis was conducted to discern relationships between variables, with a significance level (α) set at 0.05. Model 6 in Hayes’s (2013) PROCESS macro for chain mediation analysis to examine the mediating role of executive function and prospective memory. Additionally, we conducted moderated mediation analysis by employing Model 92 in PROCESS macro to establish whether sex moderated the indirect paths and direct path.

## 3 Results

### 3.1 General demographic data of subjects

Here, 713 participants [363 males (50.9%) and 350 females (49.1%)] completed the survey. For age distribution, 226 (31.7%), 184 (25.8%), 128 (18.0%), 132 (18.5%), and 43 (6.0%) respondents were aged 18–24 years, 25–34 years, 35–44 years, 45–54 years, and > 55 years, respectively. Table 1 shows the other general demographic data.

**Table 1:** General demographic data of subjects (*n* = 713).

	Variables	Number	Percentage (%)
Total		713	100
Sex	Male	363	50.90
	Female	350	49.10
Age	18–24	226	31.70
	25–34	184	25.80
	35–44	128	18.00
	45–54	132	18.50
	≥55	43	6.00
Education	Elementary school	25	3.50
	Junior high school	58	8.10
	High school or technical secondary school	70	9.80
	Junior college	168	23.60
	College	327	45.90
	College above	65	9.10
Marriage	Not married	406	56.90
	Married	255	35.80
	Divorced	46	6.50
	Widowed	6	0.80
Residence	Country	151	21.20
	Town	252	35.30
	City	310	43.50

### 3.2 The test of common method bias

Questionnaires with good reliability and validity were used as measuring instruments to control the bias effect of common methods. The confidentiality of the results was emphasized in the test process, and some questionnaire items were scored using the reverse scoring approach.

The common method was examined using the Harman single-factor test (24, 25). The exploratory factor analysis revealed that there were 35 factors without rotation, and the variance interpretation percentage of the first principal component was <40% (22.221%), indicating that no significant common method bias was found in the measurement.

### 3.3 Confirmatory factor analysis

Before testing the hypothesis, we used confirmatory factor analysis (CFA) to validate the measurement model. The measurement model includes four potential factors: AUDIT, BRIEF-A, PRM, and BIS-11. The CFA results of this study are shown in Table 2. Results showed that the data of the five-factor model were in good fit [χ2 (1763) = 2546.870, values of CFI = 0.925, TLI = 0.922, SRMR = 0.045, RMSEA = 0.027]. These findings substantiate that the model’s goodness of fit surpasses that of other factor models significantly. The results from the CFAs strongly affirm the discriminant validity of our study instruments.

**Table 2:** Results of confirmatory factors analysis.

Models	Variables	χ^2^	df	χ^2^/df	CFI	TLI	SRMR	RMSEA
Four-factor model	AUDIT, BRIEF-A, PRM, BIS-11	2546.870	1763	1.444	0.925	0.922	0.045	0.027
Three-factor model	AUDIT, BRIEF-A + PRM, BIS-11	3215.701	1766	1.821	0.861	0.856	0.051	0.037
Two-factor model	AUDIT+BRIEF-A, PRM + BIS-11	3499.082	1768	1.979	0.834	0.829	0.055	0.040
Single-factor model	AUDIT+BRIEF-A + PRM + BIS-11	3987.748	1769	2.254	0.788	0.781	0.055	0.045

AUDIT, Alcohol Use Disorders Identification Test; PRM, Prospective and Retrospective Memory Questionnaire; BRIEF-A, Behavior Rating Inventory of Executive Function-Adult Version; BIS-11, Barratt Impulsiveness Scale-11.

### 3.4 Comparison of different AUDIT groups between male and female

**Figure 1:**
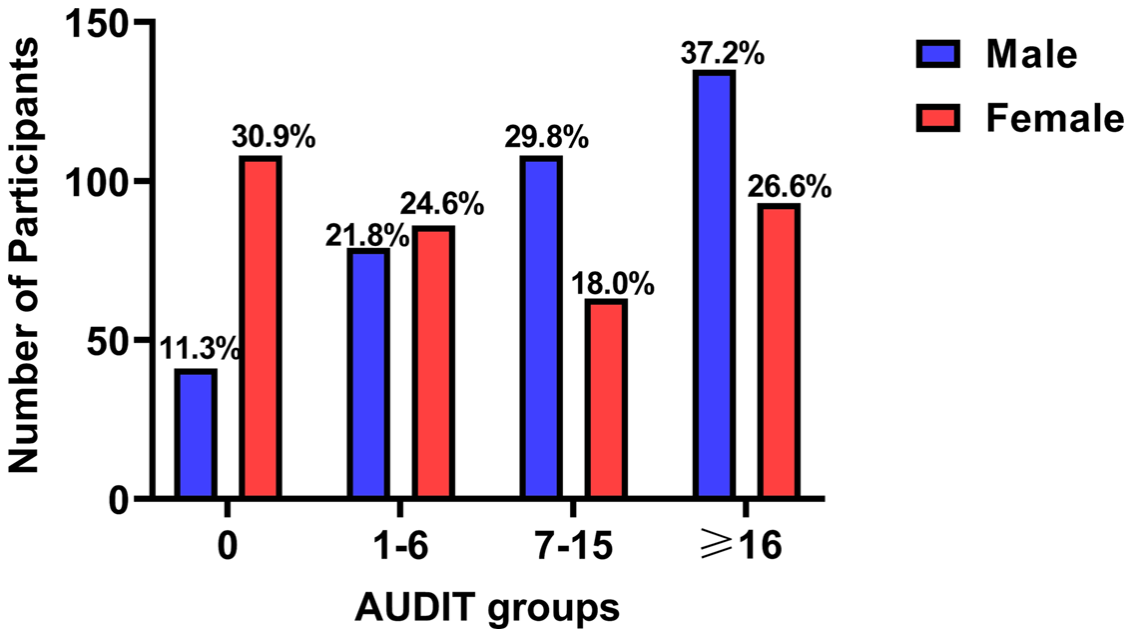
Comparison of different AUDIT groups between male and female. AUDIT, Alcohol Use Disorders Identification Test.

As shown in Figure 1, 11.3% of males and 30.9% of females were in the AUDIT = 0 group (no alcohol consumption). The AUDIT = 1–6 group (low risk drinking) included 21.8% of males and 24.6% of females. The AUDIT = 7–15 group (risky/high risk drinking) included 29.8% of males and 18.0% of females. The AUDIT ≥16 group (alcohol dependence) included 37.2% of males and 26.6% of females. Furthermore, males were significantly higher than females in total AUDIT scores (Ζ = −6.435, *p*<0.001). It might be indicated that there is a higher proportion of females who do not consume alcohol, and when the degree of alcohol consumption rises, the proportion of females declines while the number of males rises.

### 3.5 Comparisons of the PRM, BRIEF-A, and BIS-11 scores among the different AUDIT groups (median [IQR])

The results showed that the BRIEF-A and BIS-11 scores tended to increase gradually, while PRM tended to decrease gradually with the increase in AUDIT scores (*p* < 0.001; Table 3).

**Table 3:** Comparisons of the PRM, BRIEF-A, and BIS-11 scores among different AUDIT groups (Median [IQR]).

	Sex	AUDIT = 0 (*N* = 149)	AUDIT = 1–6 (*N* = 165)	AUDIT = 7–15 (*N* = 171)	AUDIT≥16 (*N* = 228)	*Z*	*p*	*Post hoc*
PRM scores	Male	66 (56, 68)	66 (56, 73)	46 (41,61)	43 (40, 47)	121.326	<0.001***	1 = 2>3>4
	Female	61 (53, 70)	59 (52, 66)	43 (41, 49)	42 (39, 48)	156.787	<0.001***	1 = 2>3 = 4
	Total	62 (54, 70)	62 (54, 72)	45 (41,54)	43 (40,47)	267.329	<0.001***	1 = 2>3>4
BRIEF-A scores	Male	108 (83, 137)	97 (80, 131)	134 (108, 140)	138 (130, 142)	63.336	<0.001***	4>3>2 = 1
	Female	102 (80, 126)	97 (86, 130)	138 (134, 142)	138 (134, 142)	139.779	<0.001***	4 = 3>2 = 1
	Total	102 (81, 128)	97 (85, 130)	136 (113, 141)	138 (132,142)	196.757	<0.001***	4>3>2 = 1
BIS-11 scores	Male	76(57,84)	68 (58, 82)	90 (70, 97)	93 (87, 98)	93.793	<0.001***	4>3>2 = 1
	Female	73 (64, 85)	76(66,86)	93 (86, 99)	94 (88, 100)	134.952	<0.001***	4 = 3>2 = 1
	Total	74 (64, 84)	73 (61, 84)	91 (78, 98)	93 (87, 99)	211.810	<0.001***	4>3>2 = 1

AUDIT, Alcohol Use Disorders Identification Test; AUDIT = 0 means no alcohol consumption; AUDIT = 1–6 means low risk drinking; AUDIT = 7–15 means risky/high risk drinking; AUDIT ≥ 16 means alcohol dependence; PRM, Prospective and Retrospective Memory Questionnaire; BRIEF-A, Behavior Rating Inventory of Executive Function-Adult Version; BIS-11, Barratt Impulsiveness Scale-11. *p < 0.05, **p < 0.01, ***p < 0.001. The median scores and IQR were round up to the nearest whole number.

### 3.6 Sex differences in PRM, BRIEF-A, and BIS-11 scores among different AUDIT groups (median [IQR])

As indicated in Table 4, while there wasn’t a significant overall difference between males and females in PRM, BRIEF-A, and BIS scores (*p* > 0.05), male PRM scores were significantly higher than those of females in the AUDIT = 1–6 group (*p* < 0.01), and male BIS-11 scores were significantly lower than those of females in the AUDIT = 7–15 group (*p* < 0.05). Furthermore, male BRIEF-A scores were significantly lower than those of females in both the AUDIT = 1–6 and AUDIT = 7–15 groups (*p* < 0.05). This suggests a potential association between alcohol use and more severe executive dysfunction, prospective memory impairment, and impulsive behavior in females compared to males.

**Table 4:** Sex differences in PRM, BRIEF-A, and BIS-11 scores among different AUDIT groups (Median [IQR]).

		Sex	Median (IQR)	*Z*	*p*
AUDIT = 0	PRM scores	Male	63 (55, 68)	−0.136	0.892
		Female	61 (53, 70)		
	BRIEF-A scores	Male	103 (82, 138)	−0.572	0.567
		Female	102 (80, 126)		
	BIS-11 scores	Male	76 (60, 84)	−0.096	0.924
		Female	73 (64, 85)		
AUDIT = 1–6	PRM scores	Male	66 (56, 73)	−3.059	<0.01
		Female	59 (52, 66)		
	BRIEF-A scores	Male	97 (80, 131)	−0.728	0.467
		Female	97 (86, 130)		
	BIS-11 scores	Male	68 (58, 82)	−3.200	<0.01
		Female	76 (66, 86)		
AUDIT = 7–15	PRM scores	Male	46 (41, 61)	−1.930	0.054
		Female	43 (41, 49)		
	BRIEF-A scores	Male	134 (108, 140)	−2.550	<0.05
		Female	138 (134, 142)		
	BIS-11 scores	Male	90 (70, 96.75)	−2.300	<0.05
		Female	93 (86, 99)		
AUDIT≥16	PRM scores	Male	43 (40, 47)	−0.924	0.356
		Female	42 (39, 48)		
	BRIEF-A scores	Male	138 (130, 142)	−0.787	0.431
		Female	138 (134, 142)		
	BIS-11 scores	Male	93 (87, 98)	−1.757	0.079
		Female	94 (88, 100)		
Total	PRM scores	Male	49 (42, 65)	−0.670	0.497
		Female	51 (43, 62)		
	BRIEF-A scores	Male	132 (100, 140)	−1.667	0.096
		Female	130 (93, 139)		
	BIS-11 scores	Male	87 (70, 94)	−0.348	0.728
		Female	86 (72, 94)		

AUDIT, Alcohol Use Disorders Identification Test; AUDIT = 0 means no alcohol consumption; AUDIT = 1–6 means low risk drinking; AUDIT = 7–15 means risky/high risk drinking; AUDIT ≥ 16 means alcohol dependence; PRM: Prospective and Retrospective Memory Questionnaire; BRIEF-A: Behavior Rating Inventory of Executive Function-Adult Version; BIS-11: Barratt Impulsiveness Scale-11. The median scores and IQR were round up to the nearest whole number.

### 3.7 Comparisons of AUDIT, PRM, BRIEF-A, and BIS-11 scores among different demographic characteristics

There were significant differences in AUDIT, PRM, and BIS-11 scores among different age, education, marriage, and residence groups, as well as in BRIEF-A scores among different marriage and residence groups (Table 5).

**Table 5:** Comparisons of AUDIT, PRM, BRIEF-A, and BIS-11 scores among different demographic characteristics (Median [IQR]).

		AUDIT	PRM	BRIEF-A	BIS-11
Age	18–24	4 (1, 14)	55 (44, 67)	128 (92, 139)	84 (68, 92)
	25–34	12 (0, 18)	48 (42, 62)	132 (92, 139)	87(72, 94)
	35–44	12 (1, 17)	48 (43, 61)	133 (102, 138)	88(72, 97)
	45–54	14 (5, 18)	49 (42, 61)	132 (97, 140)	87 (67, 96)
	≥55	15 (3, 20)	47 (40, 56)	134 (107, 145)	89 (83, 95)
*Z*		25.591	17.001	6.221	9.891
*p*		<0.001	<0.01	0.183	<0.05
Education	Elementary school	12 (2, 20)	48 (42, 57)	134 (114, 141)	88 (77, 96)
	Junior high school	7 (0, 17)	48 (40, 63)	132 (93, 139)	88 (70, 97)
	High school or technical secondary school	13 (3, 17)	48 (42, 63)	132 (105, 138)	88 (75, 94)
	Junior college	14 (1, 19)	48(42,58)	134 (99, 139)	90 (74, 95)
	College	7 (1, 16)	52 (43, 66)	129 (92, 139)	84 (67, 93)
	College above	11 (1, 18)	51 (44, 67)	131 (93, 140)	81 (66, 96)
*Z*		10.051	14.167	6.221	12.817
*p*		0.074	<0.05	0.285	<0.05
Marriage	Not married	3 (0, 14)	57 (45, 67)	125 (89, 139)	81 (66, 92)
	Married	12 (1, 17)	49 (42, 61)	131 (96, 139)	87 (72, 95)
	Divorced	19 (13, 22)	43 (40, 47)	139 (135, 145)	94 (90, 102)
	Widowed	16 (12, 20)	42 (39, 50)	143 (120, 150)	88 (76, 102)
*Z*		61.553	50.002	37.125	38.722
*p*		<0.001	<0.001	<0.001	<0.001
Residence	Country	14 (6, 20)	44 (41, 51)	136 (127, 141)	91 (84, 99)
	Town	13 (1, 18)	48 (42, 60)	134 (102, 140)	89 (74, 97)
	City	4 (1, 15)	57 (46, 68)	115 (85, 137)	79 (66, 91)
*Z*		33.083	63.250	44.340	60.429
*p*		<0.001	<0.001	<0.001	<0.001

AUDIT, Alcohol Use Disorders Identification Test; PRM, Prospective and Retrospective Memory Questionnaire; BRIEF-A, Behavior Rating Inventory of Executive Function-Adult Version; BIS-11, Barratt Impulsiveness Scale-11. The median scores and IQR were round up to the nearest whole number.

### 3.8 Correlation analysis

A partial correlation analysis of the variables controlling for age, education, marriage, and residence was performed to exclude the interference of demographic variables. Table 6 shows the median scores, interquartile range, and correlation values of all the observed variables. Sex exhibited a positive correlation with PRM and BIS-11, while displaying a negative correlation with AUDIT and BRIEF-A. This implies that females tend to have higher scores in PRM and BIS-11, and lower scores in AUDIT and BRIEF-A compared to males. On the other hand, AUDIT was positively correlated with BRIEF-A and BIS-11 and negatively correlated with PRM. Additionally, PRM was negatively correlated with BRIEF-A and BIS-11. Finally, BRIEF-A was positively correlated with BIS.

**Table 6:** Partial correlation analysis on Sex, AUDIT, PRM, BRIEF-A, and BIS-11, respectively (Median [IQR]).

	Median [IQR]	1	2	3	4	5
1 Sex^a^	0 (0, 1)	1				
2 AUDIT	11 (1, 17)	−0.24**	1			
3 PRM	50 (43, 64)	0.03	−0.52***	1		
4 BRIEF-A	131 (95, 139)	−0.06	0.49***	−0.68***	1	
5 BIS-11	86 (70, 94)	0.02	0.47***	−0.64***	0.72***	1

a Sex is a dummy variable, Male = 0, Female = 1.

AUDIT, Alcohol Use Disorders Identification Test; PRM, Prospective and Retrospective Memory Questionnaire; BRIEF-A, Behavior Rating Inventory of Executive Function-Adult Version; BIS-11, Barratt Impulsiveness Scale-11. *p < 0.05, **p < 0.01, ***p < 0.001. The median scores and IQR were round up to the nearest whole number.

### 3.9 Testing for the chain mediation model

This study explored the intermediary role of BRIEF-A and PRM between AUDIT and BIS-11, with age, education, marriage, and residence as the control variables. The deviation correction method (with 5,000 bootstraps) was used to obtain a 95% Confidence Interval (CI) to test the significance of the effects. The statistical outcome was considered significant if the CI did not contain 0. In this regard, AUDIT had significant predictive effects on BRIEF-A, PRM, and BIS-11 (Table 7). Moreover, BRIEF-A was significantly predictive of PRM and BIS-11, and PRM had significant predictive effects on BIS-11. Table 8 shows the mediating effects, direct effects, and corresponding effect scales, and they indicate that BRIEF-A and PRM act as intermediaries between AUDIT and BIS-11.

**Table 7:** The chain-mediation analysis of BRIEF-A and PRM between AUDIT and BIS-11.

Predictors	Model 1 (BRIEF-A)			Model 2 (PRM)			Model 3 (BIS-11)		
	*β*	SE	*t*	*β*	SE	*t*	*β*	SE	*t*
Age	−0.38	0.76	−0.50	0.12	0.31	0.40	−0.26	0.37	−0.71
Education	−0.04	0.72	−0.06	0.41	0.29	1.41	−0.61	0.35	−1.76
Marriage	0.81	1.37	0.59	1.21	0.55	2.18*	−0.50	0.66	−0.75
Residence	−4.60	1.09	−4.21***	1.49	0.45	3.35***	−1.12	0.54	−2.09*
AUDIT	1.47	0.10	15.03***	−0.37	0.05	−8.16***	0.16	0.06	2.87**
BRIEF-A				−0.27	0.02	−18.11***	0.32	0.02	14.62***
PRM							−0.32	0.05	−7.09***
*R* ^2^			0.30			0.55			0.60
*F*			61.78			145.33			152.89

AUDIT, Alcohol Use Disorders Identification Test; PRM, Prospective and Retrospective Memory Questionnaire; BRIEF-A, Behavior Rating Inventory of Executive Function-Adult Version; BIS-11, Barratt Impulsiveness Scale-11. *p < 0.05, **p < 0.01, ***p < 0.001.

**Table 8:** The 95% Confidence Interval (CI) of the mediating effect test and deviation corrections.

	Effect value	95% confidence interval		Effect ratio
		LLCI	ULCI	
Total effects	0.47	0.41	0.54	
Direct effects	0.09	0.03	0.15	18.57%
AUDIT→BRIEF-A → BIS-11	0.25	0.20	0.31	53.59%
AUDIT→PRM → BIS-11	0.06	0.04	0.09	13.50%
AUDIT→BRIEF-A → PRM → BIS-11	0.07	0.05	0.10	14.56%
Indirect effects	0.39	0.33	0.45	81.65%

AUDIT, Alcohol Use Disorders Identification Test; PRM, Prospective and AUDIT: Alcohol Use Disorders Identification Test; PRM, Prospective and Retrospective Memory Questionnaire; BRIEF-A, Behavior Rating Inventory of Executive Function-Adult Version; BIS-11, Barratt Impulsiveness Scale-11.

### 3.10 Testing for the moderated mediation model

The AUDIT-Sex interaction had significant effects on BRIEF-A (β = 0.1305, *p* < 0.05) and BIS-11 (β = 0.138, *p* < 0.05) (Table 9). Similarly, the BRIEF-A-Sex and the PRM-Sex interactions had significant effects on PRM (β = 0.126, *p* < 0.05) and BIS-11 (β = 0.206, *p* < 0.05), respectively. These findings indicate that sex influenced the associations between AUDIT and BRIEF-A, AUDIT and BIS-11, BRIEF-A and PRM, and PRM and BIS-11.

**Table 9:** Regression results of moderated mediation.

Predictors	Model 1 (BRIEF-A)			Model 2 (PRM)			Model 3 (BIS-11)		
	*β*	SE	*t*	*β*	SE	*t*	*β*	SE	*t*
AUDIT	0.523	0.04	15.18***	−0.283	0.03	−8.82***	0.136	0.03	4.34***
Sex	0.130	0.07	1.98*	−0.147	0.05	−2.79**	0.179	0.05	3.65***
Age	−0.008	0.03	−0.258	0.002	0.02	0.10	−0.004	0.02	−0.18
Education	−0.002	0.03	−0.060	0.027	0.02	1.20	−0.029	0.02	−1.40
Marriage	0.040	0.05	0.76	0.074	0.04	1.72	−0.002	0.04	−0.05
Residence	−0.161	0.04	−3.82***	0.098	0.03	2.85**	−0.045	0.03	−1.39
BRIEF-A				−0.532	0.03	−17.46***	0.488	0.03	14.40***
PRM							−0.232	0.04	−6.63***
Sex* AUDIT	0.131	0.07	2.00*	−0.088	0.06	−1.40	0.14	0.06	2.22*
Sex* BRIEF-A				0.126	0.06	2.07*	−0.11	0.07	−1.69
Sex* BRI							0.206	0.07	2.97**
R^2^		0.31			0.56			0.62	
*F*		45.59			99.34			105.25	

AUDIT, Alcohol Use Disorders Identification Test; PRM, Prospective and Retrospective Memory Questionnaire; BRIEF-A, Behavior Rating Inventory of Executive Function-Adult Version; BIS-11, Barratt Impulsiveness Scale-11. *p < 0.05, **p < 0.01, ***p < 0.001.

**Figure 2:**
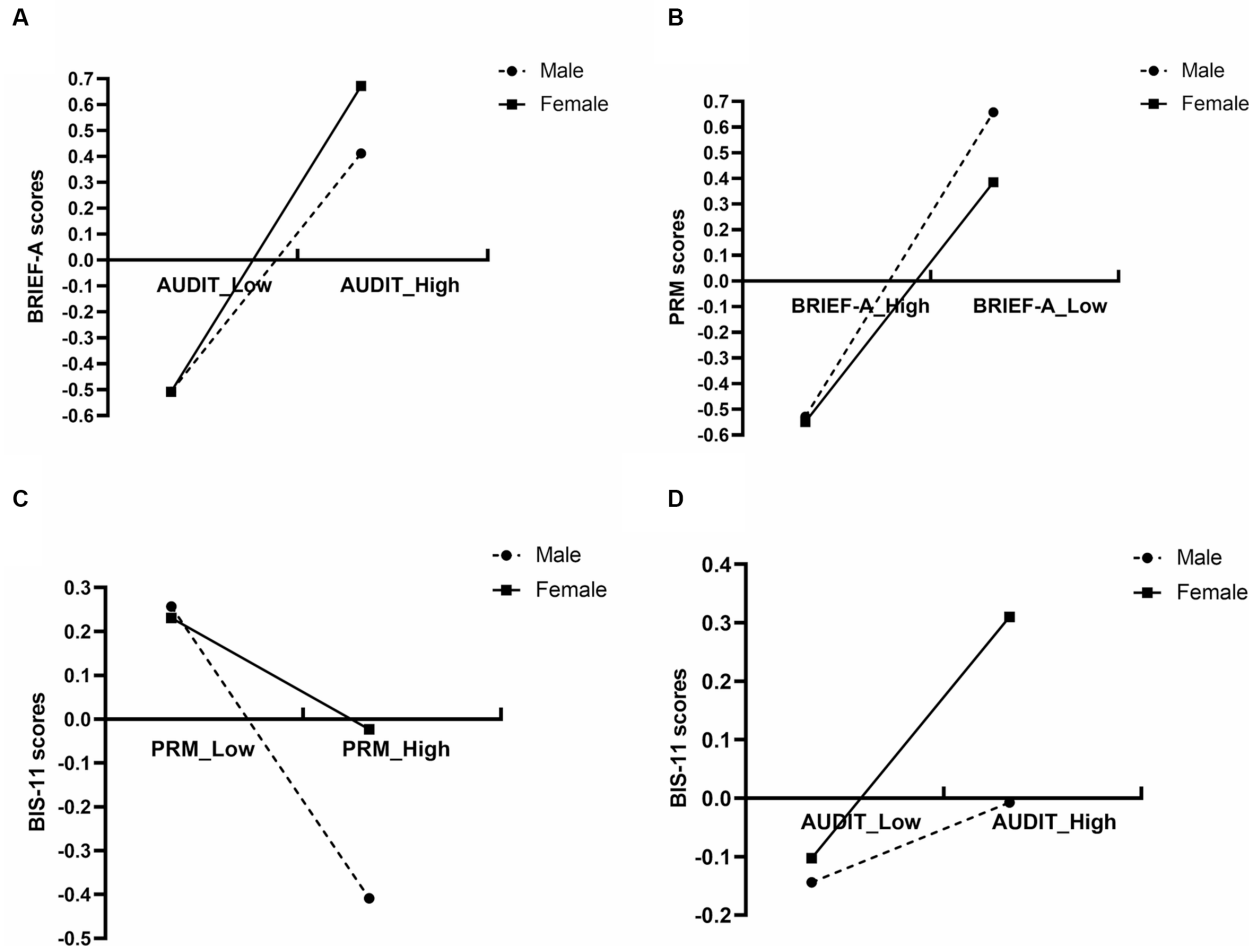
**(A)** The simple slope test of the moderator effect of sex on the relationship between BRIEF-A and PRM. **(B)** The simple slope test of the moderator effect of sex on the relationship between PRM and BIS-11. **(C)** The simple slope test of the moderator effect of sex on the relationship between AUDIT and BIS-11. **(D)** The simple slope test of the moderator effect of sex on the relationship between AUDIT and BRIEF-A. AUDIT, Alcohol Use Disorders Identification Test; BRIEF-A, Behavior Rating Inventory of Executive Function-Adult Version; PRM, Prospective and Retrospective Memory Questionnaire; BIS-11, Barratt Impulsiveness Scale-11.

Additionally, simple slope analyses were conducted to illustrate these significant interactions and explore whether the male slopes differed from those of females in the four models. The results showing the relationships between AUDIT and BRIEF-A, AUDIT and BIS-11, BRIEF-A and PRM, and PRM and BIS-11 in different sexes were plotted in Figures 2A–D. Specifically, AUDIT was positively correlated with BRIEF-A, and the influence of AUDIT on BRIEF-A in females (β = 0.590, *t* = 12.08, *p* < 0.001) was stronger than in males (β = 0.459, t = 9.96, *p* < 0.001) (Figure 2A). Additionally, BRIEF-A was negatively correlated with PRM, and the influence of BRIEF-A on PRM in males (β = −0.593, t = −14.95, *p* < 0.001) was stronger than in females (β = −0.467, *t* = −10.11, *p* < 0.001) (Figure 2B). Furthermore, PRM was negatively correlated with BIS-11, and the influence of PRM on BIS-11 in males (β = −0.333, *t* = −6.74, *p* < 0.001) was stronger than in females (β = −0.127, *t* = −2.58, *p* < 0.05) (Figure 2C). Finally, AUDIT was not significantly linked with BIS-11 (β = 0.207, *t* = 1.69, *p* = 0.09) in males but was positively correlated with BIS-11 (β = 0.068, *t* = 4.31, *p* < 0.001) in females (Figure 2D).

Furthermore, the analysis of conditional direct and indirect effects confirmed that, there was a significant difference between males and females in the indirect effect AUDIT→BRIEF-A → PRM → BIS; the indirect effect between AUDIT and BIS was more significant in males (β = 0.091) than in females [β = 0.035, 95%CI = (−0.106, −0.009)] (Table 10).

**Table 10:** Bootstrapped conditional direct and indirect effects.

	Direct effect and mediating effect on male and female groups					Index			
	Sex	*β*	BootSE	LLCI	ULCI	Index	BootSE	LLCI	ULCI
Direct effect	Male	0.0683	0.04	−0.011	0.148				
	Female	0.207	0.05	0.113	0.301				
Indirect effect 1	Male	0.250	0.04	0.178	0.334	0.004	0.054	−0.099	0.110
	Female	0.254	0.04	0.187	0.327				
Indirect effect 2	Male	0.080	0.02	0.042	0.127	−0.038	0.028	−0.097	0.013
	Female	0.042	0.02	0.008	0.077				
Indirect effect 3	Male	0.091	0.02	0.057	0.133	−0.056	0.025	−0.106	−0.009
	Female	0.035	0.02	0.007	0.069				

Indirect effect 1: AUDIT→BRIEF-A → BIS-11. Indirect effect 2: AUDIT→PRM→BIS. Indirect effect 3: AUDIT→BRIEF-A → PRM→BIS. AUDIT, Alcohol Use Disorders Identification Test; PRM, Prospective and Retrospective Memory Questionnaire; BRIEF-A, Behavior Rating Inventory of Executive Function-Adult Version; BIS-11, Barratt Impulsiveness Scale-11.

## 4 Discussion

Impulsivity is related to behavioral inhibition and encompasses a wide range of actions associated with an individual’s ability to suppress thoughts or actions appropriately (9). In this regard, addictive behaviors like substance misuse reflect multiple impulsive choices. A previous study reported that AUD patients performed worse than the control group in all the impulsive behavioral tests (26). Furthermore, growing data from separate lines of research suggest that impulsivity is associated with relapse in alcohol consumption (27). Several studies have also reported that impulsivity is associated with various socially risky behaviors (28–30). Therefore, studying the psychological mechanism of impulsivity in those with AUD or at risk for AUD is critical for managing alcohol addiction and preventing social harm.

### 4.1 EF and PM may play a chain intermediary role between alcohol use and impulsivity

Herein, we discovered that impulsivity increased with the severity of alcohol use, as did executive dysfunction and PM impairment, implying that impulsivity in those with AUD or at risk for AUD correlates with both executive dysfunction and PM impairment. Furthermore, after controlling for variables such as age, education level, marital status, and place of residence, mediation analysis indicated that EF and PM may mediate between alcohol use and impulsivity. We detail how previous relevant research supports our mediation model in this section.

#### 4.1.1 Alcohol use is associated with executive dysfunction

As a higher-order cognitive ability, EF comprises several tenets, including attention, perseveration, goal-orientation, planning, problem-solving, and working memory (31, 32). These abilities allow individuals to organize and change their behavior to achieve set goals (33). A previous study (34) revealed that alcohol-related executive function deficits encompass each EF subcomponent. Furthermore, a systematic review (35), indexed ‘Studies published from 2012 to 2022’, concluded that excessive alcohol consumption caused frontal lobe damage and that changes in prefrontal white-matter pathways underlie executive dysfunction in AUD patients.

#### 4.1.2 Alcohol use is associated with PM impairment

A PM is the cognitive capacity to recall and execute a task at a designated time in the future (36). Studies investigating the impact of excessive alcohol use on PM have consistently found that excessive drinkers reported more lapses in various aspects of their everyday PM than low-dose alcohol users or non-users (37, 38). Leitz et al. discovered that alcohol use acutely caused global impairments across all (regular, irregular, event-based, and time-based) PM tasks (39) and that future event simulation (a PM training method) significantly improved PM performance on these tasks and attenuated the acute alcohol consumption-induced PM deficit (40, 41).

#### 4.1.3 EF and PM may play a chain intermediary role between alcohol dependence and impulsivity

A previous study reported that impulsivity is mediated by specific EF components, such as behavioral flexibility, behavioral inhibition, planning, and so on (42). Therefore, executive dysfunction might be associated with impulsive behavior. According to research, poor EF increases the likelihood of healthy young adults engaging in risky and potentially dangerous acts (28). Koob and Volkow (43) proposed that excessive alcohol consumption may promote EF deficits by dysregulating glutamatergic, GABAergic, and Dopaminergic (DA) neuronal networks in the prefrontal cortex, which perpetuates the dysregulation of reward and stress function and induces impulsive drug use.

Furthermore, EF is involved in PM formation. A previous study reported that the four phases of the PM process (intention formation, intention retention, intention reinstantiation, and intention execution) are assumed to require different amounts of executive processing, most of which is demanded in the intention formation and intention execution phases and that EFs are related to PM performance across a range of prospective paradigms (44). Some studies reported that substance misusers have impaired PM attributable to central executive deficits caused by the substance misuse-associated frontal lobe damage (45).

Moreover, some studies have confirmed that PM may affect impulsive behavior. For example, PM performance was reported to be negatively associated with impulsivity (12). By adding weights to delayed benefits, Episodic Future Thinking (EFT) has been confirmed as an effective method to counter impulsive behavior and decisions (46–48). It has been reported that damage to the brain area responsible for EFT impairs the use of EFT in reward-based decision-making (49).

In summary, our study’s mediation model implies that alcohol consumption may be associated with impulsivity formation through executive dysfunction and PM impairment. Recognition of the relationship between EF, PM, and impulsivity may inform the scientific inquiry into behavioral problems in those with AUD or at risk for AUD. It suggests that impulsivity in those with AUD or at risk for AUD may be treated by improving EF and PM.

### 4.2 Sex factors play a moderating role in the alcohol use and impulsivity mediating model

This study found that alcohol use levels among female participants were significantly lower than in males. However, the female alcohol users had more severe executive dysfunction, PM impairment, and impulsivity than the males. Furthermore, previous research found no difference in EF (50) and PM (51–53) between normal males and females. Therefore, the more severe executive dysfunction, PM impairment, and impulsivity in females with AUD [or who report risky drinking] may be attributed to the physiological susceptibility of the females to alcohol consumption.

As of now, we have not come across specific research reports addressing sex differences in executive dysfunction and prospective memory impairment related to alcohol use. However, in line with our own findings, a substantial body of research suggests that AUD is more likely to lead to both physiological and psychological repercussions in females.

Firstly, according to research, progress from initiation of substance use to the onset of physical and psychological health complications is more rapid and severe among females with Substance Use Disorder (SUD) (54). Compared to their male counterparts, females develop comparable or more pronounced alcohol-related liver and cardiovascular diseases at lower alcohol consumption levels and are also more vulnerable to brain damage and related cognitive impairments (55). AUD generally leads to the development of health complications much more rapidly in females than in males. For example, cirrhosis, alcohol-induced cardiomyopathy, and peripheral neuropathy develop in females after fewer years of heavy drinking than in males (56).

Secondly, females are more vulnerable to brain damage and neurotoxic effects of alcohol than males (56, 57). Compared to non-drinking individuals, both males and females with AUD show reduced brain volume, but brain shrinkage and cognitive dysfunction appear to develop much more quickly in females than in males (56, 57). Additionally, females with AUD may experience a greater short-term memory impairment than their male counterparts (57). In a previous study, it was found that females showed larger total cerebellar brain volume than males at any given alcohol consumption level and that the females were much more susceptible to the undesirable effects of alcohol (58). Therefore, the sex-based differences in brain volume between females and males may be interpreted as causing a much faster alcohol absorption rate in females than males (58). The above study explains the neurophysiological mechanism of alcohol in causing more severe executive dysfunction, PM impairment, and impulsive behavior in females than in males.

Moreover, this investigation revealed that the indirect influence of EF and PM in the relationship between AUDIT scores and BIS scores was more pronounced in males than females (Table 10). In summary, the direct impact is more robust in the female group, whereas the mediating effect is more pronounced in the male group. The influence of executive dysfunction on male PM outweighed that on female PM (Figure 2B), and the effect of PM impairment on impulsive behavior in males exceeded that in females (Figure 2C). This implies that enhancing executive dysfunction in males may be more likely to enhance PM, while ameliorating PM impairment may be more likely to mitigate impulsive behavior.

**Figure 3:**
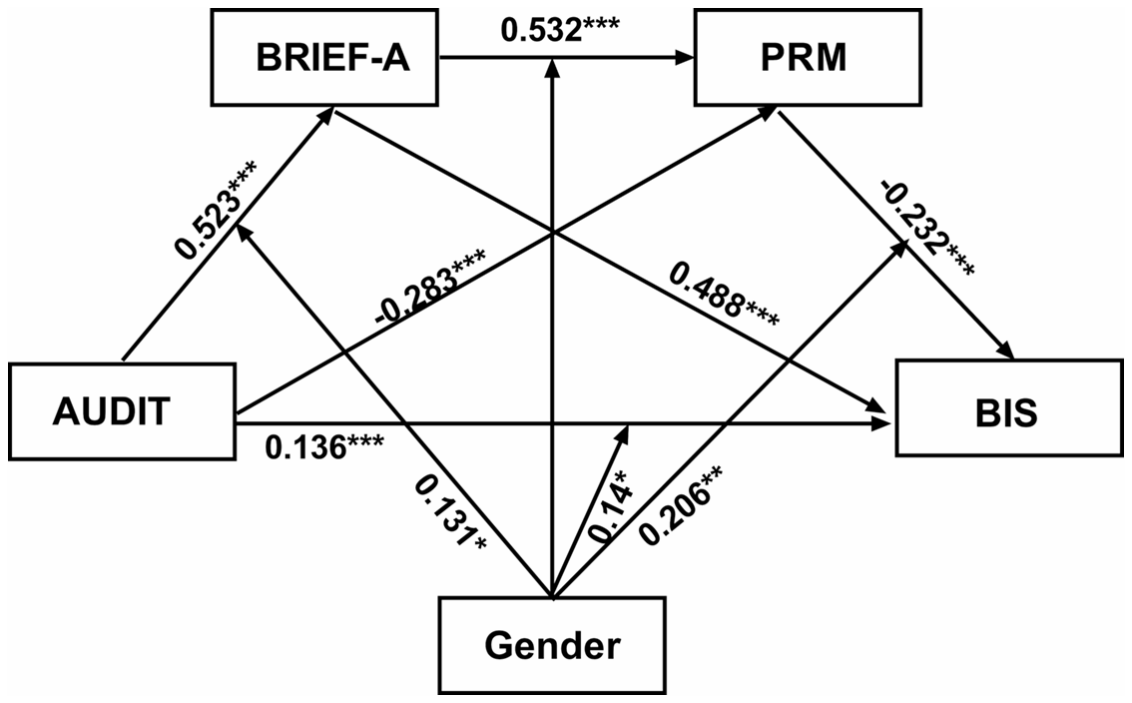
The hypothetical moderated mediation model. AUDIT, Alcohol Use Disorders Identification Test; PRM, Prospective and Retrospective Memory Questionnaire; BRIEF-A, Behavior Rating Inventory of Executive Function-Adult Version; BIS-11, Barratt Impulsiveness Scale-11.

To conclude, this study suggests that alcohol consumption may contribute to the development of impulsivity through the pathways of executive dysfunction and PM impairment. Even though the prevalence of alcohol use among females is lower than that of males, it is associated with more pronounced executive dysfunction, PM deficits, and impulsive behavior in females. Furthermore, the study proposes that the indirect impact of EF and PM in the relationship between AUDIT scores and BIS scores is more prominent in males than females. These findings provide both clinical and theoretical foundations for addressing issues related to alcohol use (see Figure 3).

## 5 Limitation

This study had some limitations. First, it relied on self-report measures of EF, PM, and impulsive behavior. Second, there were issues about polydrug use, the need to control for the co-morbidity of other conditions such as depression, and better drug screening methods, such as excluding antidepressant drug and anxiolytics. Third, we adopted a cross-sectional design, indicating that the mediating equations in this study are merely inferential and cannot provide evidence for causality. What’s more, many of the associations in the model are very weak; it may be due to the small sample size of this study and the need to further increase the sample for future studies. We therefore advise caution when interpreting this measure. Moreover, some longitudinal studies found that impulsivity was the cause of AUD (59), and therefore impulsiveness is both a cause and a consequence of AUD. Based on the risks of clinical AUD leading to impulsive conduct and the fact that impulsivity, in turn, encourages alcohol misuse, more research on alcohol use leading to impulsivity is recommended. Consequently, this study explored the mechanism by which alcohol consumption leads to impulsivity and developed mediating equations. These findings need to be validated in practice.

Further, some other research had shown that females tended to have less impulsivity and less AUD and alcohol consumption than males (60). This seemed contrary to our findings. However, the low average level of impulsivity in females might be due to the low proportion of women who drink or have AUD. Our study was more focused on showing that women’s impulsivity scores were more likely to rise compared to men’s as their AUDIT scores rise. In this study, we found that males had significantly higher AUDIT scores than females overall. Although there was no significant overall difference in BIS-11 scores between males and females, females had significantly higher BIS-11 scores when AUDIT = 1–6 and AUDIT = 7–15. Interestingly, there was no difference in BRIEF-A, PRM and BIS-11 between males and females in the AUDIT≥16 group. More sophisticated and differentiated methods of detection may be required to further assess these measures in those with AUD. Therefore, further studies should employ objective measures alongside self-report measures and incorporate better controls for the use of other drugs and mood status.

## Data availability statement

The raw data supporting the conclusions of this article will be made available by the authors, without undue reservation.

## Ethics statement

The studies involving humans were approved by Bengbu Medical College Institutional Review Board. The studies were conducted in accordance with the local legislation and institutional requirements. Written informed consent for participation was not required from the participants or the participants' legal guardians/next of kin because this research was conducted online.

## Author contributions

FD: Writing – original draft. LX: Writing – original draft. JL: Writing – original draft. XL: Data curation, Writing – original draft. YZ: Investigation, Writing – original draft. HL: Investigation, Writing – original draft. ZW: Investigation, Writing – original draft. XS: Software, Writing – original draft. JW: Investigation, Writing – original draft. JC: Data curation, Writing – original draft. YW: Software, Writing – original draft. JZ: Writing – review & editing. XZ: Writing – review & editing. DJ: Writing – review & editing.

## References

[1] Radke AK Sneddon EA Frasier RM Hopf FW. Recent perspectives on sex differences in compulsion-like and binge alcohol drinking. Int J Mol Sci. (2021) 22:3788. doi: 10.3390/ijms22073788, PMID: 33917517 PMC8038761

[2] McHugh RK Votaw VR Sugarman DE Greenfield SF. Sex and gender differences in substance use disorders. Clin Psychol Rev. (2018) 66:12–23. doi: 10.1016/j.cpr.2017.10.012, PMID: 29174306 PMC5945349

[3] Levine OB Skelly MJ Miller JD Rivera-Irizarry JK Rowson SA DiBerto JF. The paraventricular thalamus provides a polysynaptic brake on limbic CRF neurons to sex-dependently blunt binge alcohol drinking and avoidance behavior in mice. Nat Commun. (2021) 12:5080. doi: 10.1038/s41467-021-25368-y, PMID: 34426574 PMC8382748

[4] de Wit H. Impulsivity as a determinant and consequence of drug use: a review of underlying processes. Addict Biol. (2009) 14:22–31. doi: 10.1111/j.1369-1600.2008.00129.x, PMID: 18855805 PMC3640851

[5] Crews FT Boettiger CA. Impulsivity, frontal lobes and risk for addiction. Pharmacol Biochem Behav. (2009) 93:237–47. doi: 10.1016/j.pbb.2009.04.018 19410598 PMC2730661

[6] Nederkoorn C Baltus M Guerrieri R Wiers RW. Heavy drinking is associated with deficient response inhibition in women but not in men. Pharmacol Biochem Behav. (2009) 93:331–6. doi: 10.1016/j.pbb.2009.04.015, PMID: 19409923

[7] Townshend JM Duka T. Binge drinking, cognitive performance and mood in a population of young social drinkers. Alcohol Clin Exp Res. (2005) 29:317–25. doi: 10.1097/01.ALC.0000156453.05028.F5, PMID: 15770105

[8] Rupp CI Beck JK Heinz A Kemmler G Manz S Tempel K. Impulsivity and alcohol dependence treatment completion: is there a neurocognitive risk factor at treatment entry? Alcohol Clin Exp Res. (2016) 40:152–60. doi: 10.1111/acer.12924, PMID: 26683585

[9] Evenden JL. Varieties of impulsivity. Psychopharmacology. (1999) 146:348–61. doi: 10.1007/PL00005481 10550486

[10] Pattij T De Vries TJ. The role of impulsivity in relapse vulnerability. Curr Opin Neurobiol. (2013) 23:700–5. doi: 10.1016/j.conb.2013.01.023 23462336

[11] Patton JH Stanford MS Barratt ES. Factor structure of the Barratt impulsiveness scale. J Clin Psychol. (1995) 51:768–74. doi: 10.1002/1097-4679(199511)51:6<768::AID-JCLP2270510607>3.0.CO;2-1 8778124

[12] Gladwin TE Jewiss M Banic M Pereira A. Associations between performance-based and self-reported prospective memory, impulsivity and encoding support. Acta Psychol. (2020) 206:103066. doi: 10.1016/j.actpsy.2020.103066, PMID: 32247968

[13] Mioni G Stablum F McClintock SM Cantagallo A. Time-based prospective memory in severe traumatic brain injury patients: the involvement of executive functions and time perception. J Int Neuropsychol Soc. (2012) 18:697–705. doi: 10.1017/S1355617712000306, PMID: 22433779

[14] Shum DH Cahill A Hohaus LC O'Gorman JG Chan RC. Effects of aging, planning, and interruption on complex prospective memory. Neuropsychol Rehabil. (2013) 23:45–63. doi: 10.1080/09602011.2012.716761, PMID: 22973855

[15] Pavawalla SP Schmitter-Edgecombe M Smith RE. Prospective memory after moderate-to-severe traumatic brain injury: a multinomial modeling approach. Neuropsychology. (2012) 26:91–101. doi: 10.1037/a0025866 21988127 PMC3271186

[16] Ling J Heffernan TM Luczakiewicz K Stephens R. Subjective ratings of prospective memory deficits in chronic alcohol users. Psychol Rep. (2010) 106:905–17. doi: 10.2466/pr0.106.3.905-917, PMID: 20712179

[17] Lees B Meredith LR Kirkland AE Bryant BE Squeglia LM. Effect of alcohol use on the adolescent brain and behavior. Pharmacol Biochem Behav. (2020) 192:172906. doi: 10.1016/j.pbb.2020.172906, PMID: 32179028 PMC7183385

[18] Erol A Karpyak VM. Sex and gender-related differences in alcohol use and its consequences: contemporary knowledge and future research considerations. Drug Alcohol Depend. (2015) 156:1–13. doi: 10.1016/j.drugalcdep.2015.08.023, PMID: 26371405

[19] Li Q Babor TF Hao W Chen X. The Chinese translations of alcohol use disorders identification test (AUDIT) in China: a systematic review. Alcohol Alcohol. (2011) 46:416–23. doi: 10.1093/alcalc/agr012, PMID: 21467046 PMC3119458

[20] Li B. Shen Y. Zhang B. Zheng X. Wang X. Test of AUDIT in China. Chinese Mental Health Journal.. (2003) 17:1–3.

[21] Yang TX Wang Y Wang Y Su XM Ni K Lui SSY. Validity and normative data of the Chinese prospective and retrospective memory questionnaire (PRMQ) across adolescence, adults and elderly people. Memory. (2022) 30:344–53. doi: 10.1080/09658211.2021.2014526, PMID: 34919027

[22] Roth R.M. Isquith P.K. Gioia G.A. BRIEF-A: behavior rating inventory of executive function--adult version: professional manual, psychological assessment resources. (2005).

[23] Facchinetti G Piredda M Ausili D Angaroni V Albanesi B Marchetti A. Information before discharge in geriatric patients in Italy: cultural adaptation and validation of the patient continuity of care questionnaire. Eur J Ageing. (2021) 18:99–107. doi: 10.1007/s10433-020-00576-5, PMID: 33746685 PMC7925798

[24] Podsakoff PM MacKenzie SB Lee JY Podsakoff NP. Common method biases in behavioral research: a critical review of the literature and recommended remedies. J Appl Psychol. (2003) 88:879–903. doi: 10.1037/0021-9010.88.5.879, PMID: 14516251

[25] Hao Z Lirong L. Statistical remedies for common method biases. Adv Psychol Sci. (2004) 12:942–50.

[26] Rubio G Jimenez M Rodriguez-Jimenez R Martinez I Avila C Ferre F. The role of behavioral impulsivity in the development of alcohol dependence: a 4-year follow-up study. Alcohol Clin Exp Res. (2008) 32:1681–7. doi: 10.1111/j.1530-0277.2008.00746.x, PMID: 18631324

[27] Reyes-Huerta HE Dos Santos C Martinez K. Impulsive mechanisms influencing relapse in alcohol drinking. Med Hypotheses. (2018) 112:27–9. doi: 10.1016/j.mehy.2018.01.007, PMID: 29447931

[28] Reynolds BW Basso MR Miller AK Whiteside DM Combs D. Executive function, impulsivity, and risky behaviors in young adults. Neuropsychology. (2019) 33:212–21. doi: 10.1037/neu0000510, PMID: 30589284

[29] McHugh CM Chun Lee RS Hermens DF Corderoy A Large M Hickie IB. Impulsivity in the self-harm and suicidal behavior of young people: a systematic review and meta-analysis. J Psychiatr Res. (2019) 116:51–60. doi: 10.1016/j.jpsychires.2019.05.012, PMID: 31195164

[30] Dir AL Coskunpinar A Cyders MA. A meta-analytic review of the relationship between adolescent risky sexual behavior and impulsivity across gender, age, and race. Clin Psychol Rev. (2014) 34:551–62. doi: 10.1016/j.cpr.2014.08.004, PMID: 25261740

[31] Jurado MB Rosselli M. The elusive nature of executive functions: a review of our current understanding. Neuropsychol Rev. (2007) 17:213–33. doi: 10.1007/s11065-007-9040-z, PMID: 17786559

[32] Tekin S Cummings JL. Frontal-subcortical neuronal circuits and clinical neuropsychiatry: an update. J Psychosom Res. (2002) 53:647–54. doi: 10.1016/S0022-3999(02)00428-2, PMID: 12169339

[33] Banich MT. Executive function: the search for an integrated account. Curr Dir Psychol Sci. (2009) 18:89–94. doi: 10.1111/j.1467-8721.2009.01615.x

[34] Brion M D'Hondt F Pitel AL Lecomte B Ferauge M de Timary P. Executive functions in alcohol-dependence: a theoretically grounded and integrative exploration. Drug Alcohol Depend. (2017) 177:39–47. doi: 10.1016/j.drugalcdep.2017.03.018, PMID: 28554151

[35] Maharjan S Amjad Z Abaza A Vasavada AM Sadhu A Valencia C. Executive dysfunction in patients with alcohol use disorder: a systematic review. Cureus. (2022) 14:e29207. doi: 10.7759/cureus.29207 36258974 PMC9573267

[36] Brandimonte MA Einstein GO McDaniel MA. Prospective memory: Theory and applications. Psychology Press (2014).

[37] Heffernan TM. The impact of excessive alcohol use on prospective memory: a brief review. Curr Drug Abuse Rev. (2008) 1:36–41. doi: 10.2174/1874473710801010036, PMID: 19630703

[38] Heffernan T Clark R Bartholomew J Ling J Stephens S. Does binge drinking in teenagers affect their everyday prospective memory? Drug Alcohol Depend. (2010) 109:73–8. doi: 10.1016/j.drugalcdep.2009.12.013, PMID: 20071106

[39] Leitz JR Morgan CJ Bisby JA Rendell PG Curran HV. Global impairment of prospective memory following acute alcohol. Psychopharmacology. (2009) 205:379–87. doi: 10.1007/s00213-009-1546-z, PMID: 19440700

[40] Paraskevaides T Morgan CJ Leitz JR Bisby JA Rendell PG Curran HV. Drinking and future thinking: acute effects of alcohol on prospective memory and future simulation. Psychopharmacology. (2010) 208:301–8. doi: 10.1007/s00213-009-1731-0, PMID: 19967530

[41] Platt B Kamboj SK Italiano T Rendell PG Curran HV. Prospective memory impairments in heavy social drinkers are partially overcome by future event simulation. Psychopharmacology. (2016) 233:499–506. doi: 10.1007/s00213-015-4145-1, PMID: 26612619 PMC4710660

[42] Bickel WK Jarmolowicz DP Mueller ET Gatchalian KM McClure SM. Are executive function and impulsivity antipodes? A conceptual reconstruction with special reference to addiction. Psychopharmacology. (2012) 221:361–87. doi: 10.1007/s00213-012-2689-x, PMID: 22441659 PMC4035182

[43] Koob GF Volkow ND. Neurobiology of addiction: a neurocircuitry analysis. Lancet Psychiatry. (2016) 3:760–73. doi: 10.1016/S2215-0366(16)00104-8, PMID: 27475769 PMC6135092

[44] Martin M Kliegel M McDaniel MA. The involvement of executive functions in prospective memory performance of adults. Int J Psychol. (2003) 38:195–206. doi: 10.1080/00207590344000123

[45] Heffernan TM Jarvis H Rodgers J Scholey AB Ling J. Prospective memory, everyday cognitive failure and central executive function in recreational users of ecstasy. Hum Psychopharmacol. (2001) 16:607–12. doi: 10.1002/hup.349, PMID: 12404540

[46] Peters J Büchel C. Episodic future thinking reduces reward delay discounting through an enhancement of prefrontal-mediotemporal interactions. Neuron. (2010) 66:138–48. doi: 10.1016/j.neuron.2010.03.026, PMID: 20399735

[47] Benoit RG Gilbert SJ Burgess PW. A neural mechanism mediating the impact of episodic prospection on farsighted decisions. J Neurosci. (2011) 31:6771–9. doi: 10.1523/JNEUROSCI.6559-10.2011, PMID: 21543607 PMC6632845

[48] Daniel TO Stanton CM Epstein LH. The future is now: reducing impulsivity and energy intake using episodic future thinking. Psychol Sci. (2013) 24:2339–42. doi: 10.1177/0956797613488780, PMID: 24022653 PMC4049444

[49] Palombo DJ Keane MM Verfaellie M. The medial temporal lobes are critical for reward-based decision making under conditions that promote episodic future thinking. Hippocampus. (2015) 25:345–53. doi: 10.1002/hipo.22376 25284804 PMC4331231

[50] Grissom NM Reyes TM. Correction: Let’s call the whole thing off: evaluating gender and sex differences in executive function. Neuropsychopharmacology. (2019) 44:1344. doi: 10.1038/s41386-019-0367-y 30914764 PMC6785023

[51] Hsu Y-H Hua M-S. Taiwan version of the prospective and retrospective memory questionnaire: latent structure and normative data. Arch Clin Neuropsychol. (2011) 26:240–9. doi: 10.1093/arclin/acr012, PMID: 21421567

[52] Uttl B White CA Cnudde K Grant LM. Prospective memory, retrospective memory, and individual differences in cognitive abilities, personality, and psychopathology. PLoS One. (2018) 13:e0193806. doi: 10.1371/journal.pone.0193806, PMID: 29584735 PMC5870974

[53] Simard M Rouleau I Kadlec H Taler V Tuokko H Voll S. Miami prospective memory test in the Canadian longitudinal study on aging. Clin Neuropsychol. (2019) 33:137–65. doi: 10.1080/13854046.2018.1435824, PMID: 29431015

[54] Hernandez-Avila CA Rounsaville BJ Kranzler HR. Opioid-, cannabis-and alcohol-dependent women show more rapid progression to substance abuse treatment. Drug Alcohol Depend. (2004) 74:265–72. doi: 10.1016/j.drugalcdep.2004.02.001, PMID: 15194204

[55] Wilsnack SC Wilsnack RW Kantor LW. Focus on: women and the costs of alcohol use. Alcohol Res. (2013) 35:219–28. PMID: 24881330 10.35946/arcr.v35.2.12 PMC3908713

[56] Hommer DW. Male and female sensitivity to alcohol-induced brain damage. Alcohol Res Health. (2003) 27:181–5. PMID: 15303629 PMC6668882

[57] Prendergast MA. Do women possess a unique susceptibility to the neurotoxic effects of alcohol? J Am Med Womens Assoc. (1972) 59:225–7. 15354377

[58] Paul CA Au R Fredman L Massaro JM Seshadri S DeCarli C. Association of alcohol consumption with brain volume in the Framingham study. Arch Neurol. (2008) 65:1363–7. doi: 10.1001/archneur.65.10.1363, PMID: 18852353 PMC2861346

[59] Shin SH Lee S Jeon SM Wills TA. Childhood emotional abuse, negative emotion-driven impulsivity, and alcohol use in young adulthood. Child Abuse Negl. (2015) 50:94–103. doi: 10.1016/j.chiabu.2015.02.010, PMID: 25743371 PMC5356361

[60] Su J Trevino A Jamil B Aliev F. Genetic risk of AUDs and childhood impulsivity: examining the role of parenting and family environment. Dev Psychopathol. (2022) 34:1–14. doi: 10.1017/S095457942200092X 36523258

